# Association of Serum Irisin Levels with Body Composition, Metabolic Profile, Leptin, and Adiponectin Levels in Lean and Obese Children

**DOI:** 10.3390/medicina59111954

**Published:** 2023-11-05

**Authors:** Kübra Esin, Saime Batirel, Gözde Ülfer, Pakize Yigit, Nevin Sanlier

**Affiliations:** 1 Department of Nutrition and Dietetics, Faculty of Health Sciences, Tokat Gaziosmanpaşa University, Tokat 60250, Türkiye; 2 Department of Medical Biochemistry, Faculty of Medicine, Marmara University, Istanbul 34722, Türkiye; saime.batirel@marmara.edu.tr; 3 Department of Medical Biochemistry, Faculty of Medicine, Istanbul Medipol University, Istanbul 34214, Türkiye; gulfer@medipole.edu.tr; 4 Department of Statistical Analysis and Applications, Faculty of Medicine, Istanbul Medipol University, Istanbul 34810, Türkiye; pyigit@medipol.edu.tr; 5 Department of Nutrition and Dietetics, Faculty of Health Sciences, Ankara Medipol University, Ankara 06050, Türkiye; nevintekgul@gmail.com

**Keywords:** childhood obesity, irisin, adiponectin, leptin, vitamin D

## Abstract

*Background and Objectives*: Irisin is an adipo-myokine with potential metabolic effects in the body, and its association with childhood obesity is still controversial. This study aimed to investigate the relationship between serum irisin levels and anthropometric parameters, body composition, metabolic profiles, leptin, and adiponectin in obese and normal-weight children. *Methods*: The study sample consisted of a total of 80 children aged 6–10, including 44 obese children (BMI ≥ 97th percentile) and 36 normal-weight children. Anthropometric parameters were measured. Body composition was determined with the use of the Bioelectrical Impedance Analysis (BIA) method. Metabolic profiles, as well as irisin, leptin, and adiponectin levels, were analyzed. *Results*: Anthropometric parameters and body composition were found to be significantly different between the obese and normal-weight groups (*p* < 0.05). Fasting blood glucose, insulin, HOMA-IR, and leptin levels were found to be significantly higher in the obese group, while the normal-weight group had significantly higher HDL cholesterol and adiponectin levels (*p* < 0.05). Serum irisin levels did not differ between the obese and normal-weight groups, or based on sex, vitamin D levels, and insulin resistance status. There was also no statistically significant correlation between serum irisin levels and anthropometric parameters, metabolic profile, leptin, and adiponectin. *Conclusions*: The study concluded that the obese children who participated in this study had high leptin levels and low adiponectin levels, with no significant difference in the irisin levels between the groups. More comprehensive clinical studies are needed to investigate the relationship between irisin and adipokines in children.

## 1. Introduction

The prevalence of obesity is constantly increasing in both developed and developing countries [1]. This prevalence has shown more than a four-fold increase in children and adolescents in the last four decades [2]. Childhood obesity usually persists into adulthood and leads to several chronic diseases, such as insulin resistance, type 2 diabetes mellitus, dyslipidemia, hypertension, cardiovascular diseases, and non-alcoholic fatty liver disease [3]. Although the etiology of obesity is complex, one of the key factors in its development is the long-term dysregulation of energy balance, involving increased energy intake and/or decreased energy expenditure. Therefore, understanding the regulation of energy homeostasis is crucial to prevent obesity and its complications [4]. Recently, adipose tissue and skeletal muscle-derived cytokines, also known as adipokines and myokines, have gained more recognition in the prevention and treatment of obesity and its complications as they play a role in the regulation of energy metabolism [5].

Irisin is an exercise-induced adipo-myokine that regulates energy homeostasis [6]. It is a thermogenic protein that increases the synthesis of uncoupling protein 1 (UCP-1) and causes energy consumption by converting white adipose tissue into brown adipose tissue [7,8]. Given that it is a thermogenic agent, irisin has the potential to reduce obesity and its complications and improve metabolic status [9]. The relationship between irisin and obesity in children is still debated [10]. The association of irisin with anthropometric measurements and biochemical markers of obesity has been reported as positive in some studies [11,12,13,14,15], negative in some studies [16,17], and nonexistent or insignificant in others [18,19,20].

Irisin could be linked to other regulatory hormones such as adipokines, and this association may be intertwined in the maintenance of metabolic status [21]. Leptin and adiponectin are some of the main adipokines with roles in body composition, energy homeostasis, and insulin sensitivity [22]. It was reported that leptin can stimulate irisin secretion by modulating muscle metabolism, and irisin can stimulate adiponectin secretion via thermogenesis by promoting the browning of adipocytes [23]. Studies associating irisin concentrations with different adipokines in the pediatric population are limited and provide contradictory results [10]. Different studies have revealed a positive association between irisin and leptin [11,24], a negative relationship of irisin with leptin [21] and adiponectin [11], and no correlation between irisin with leptin [25] and adiponectin [24]. The reciprocal relationship between adipokines and irisin secreted from skeletal muscle tissue may have a significant function in the treatment of obesity. However, the interplay between irisin and adipokines is still scarcely described in the pediatric population [20]. 

Childhood is an important period of maturation and development in terms of behavioral habits, hormones, and body composition [17]. Due to the increasing prevalence of obesity and its serious complications, identifying potential biomarkers that can be used in determining obesity and its complications and understanding the associated mechanisms is necessary and important for prevention/treatment strategies [26,27]. This is why, in this study, we aimed to investigate the relationship between serum irisin levels and anthropometric parameters, body composition, metabolic profiles, leptin, and adiponectin in obese and normal-weight children.

## 2. Materials and Methods

### 2.1. Subjects

This case-control study included a total of 80 children aged 6–10; 46 girls and 34 boys, who were brought to the Pediatric Endocrinology Outpatient Clinic of Istanbul Medipol University Mega Hospital. This age group was selected for the sample to minimize the hormonal effects of puberty. The case group included 44 obese children (BMI ≥ 97th percentile), while the control group included 36 normal-weight children at the same ages (15th < 85th percentile). The sample included children who did not have chronic hepatic or renal diseases, had not used vitamin D, calcium, and/or multivitamin supplements in the last three months, and did not have obesity that developed due to syndromic or endocrine causes. Children who did not meet these criteria, those who did not agree to participate in the study, and those who were not Turkish were excluded. 

### 2.2. Anthropometric Measurements and Body Composition Analyses

Anthropometric measurements and body composition analyses were carried out by the researchers in person.

*Weight, height, and Body Mass Index:* Weight and height measurements of the children were taken when they were wearing light clothes and no shoes. Height was measured with the head on the Frankfurt plane, feet were touching each other at the heels, and back, buttocks, and heels in contact with the wall, using a stadiometer as children were instructed to take a deep breath. Weight was measured using an Inbody 520 brand body analysis device. Body mass index (BMI) was calculated with the formula: “weight (kg)/[height (m)]^2^” (kg/m^2^). Children with a BMI ≥ 97th percentile were considered obese, and those with a BMI = 15th < 85th percentile were considered as having normal weight [28]. Percentile values were calculated using the WHO Antro Plus program [29].

*Circumference measurements:* All measurements were taken by the researcher using an inelastic tape measure in a standardized manner. Waist circumference measurements were taken without compressing tissues, with the tape measure parallel to the floor. The child stood upright in their underwear, with both arms in a relaxed position and both legs touching, and were measured from the middle point marked between the lowest rib and the crista iliaca. Hip circumference measurements were made standing on the side while the child stood upright, from the most prominent part of the gluteal region, with the tape measure parallel to the floor [30]. Neck circumference, considered as important as waist circumference, in terms of showing the amount of upper body fat accumulation, was measured with the child standing upright, in a standardized manner, at the level of the superior edge of the cricothyroid membrane [31].

*Skinfold thickness:* To determine total body fat mass and lean body mass, skinfold thicknesses were measured using a Holtain brand skinfold caliper. The triceps skinfold was measured from the middle point between the acromion and the olecranon, with the child standing upright with arms in a relaxed position. The subscapular skinfold was measured was measured at the inferior angle of the scapula in the skinfold axis. Each measurement was taken 3 times, and the average value of the 3 measurements was recorded [30].

*Body composition analysis:* The body composition of each child (body fat mass, skeletal muscle mass, lean body mass, body water, body fat ratio) was determined using the bioelectric impedance method, using an Inbody 520 brand body analysis device. To ensure the conditions necessary for bioelectric impedance measurements, each participant did not perform intense physical activity within 24 h before the measurements, fasted for at least 4 h beforehand, did not drink water before the test, and did not have tea, coffee, or coke for at least 4 h [30].

### 2.3. Sample Collection and Laboratory Analyses

Blood samples were collected from all children who participated in the study, following 8–12 h of fasting. From the collected samples, sera were obtained using the centrifugation method. Within the same day, 25 hydroxy vitamin D, total calcium, phosphorus, alkaline phosphatase, parathyroid hormone, serum fasting blood glucose, insulin, total cholesterol, triglyceride, LDL cholesterol, and HDL cholesterol parameters were studied from the serum samples. All measurements were made at the Biochemistry Laboratories of Istanbul Medipol University Mega Hospital using commercially purchased kits, the Roche Cobas^®^ 6000 ce modular system, and the ECLIA (electrochemiluminescence immunoassay) method [32].

The measured vitamin D levels were defined to indicate “deficiency” when below 15 ng/mL, “insufficiency” when in the range of 15–19.9 ng/mL, and “normal values” when equal to or greater than 20 ng/mL [33]. In both groups, HOMA-IR (homeostasis model assessment of insulin resistance) was used as an indicator of insulin resistance, and was calculated using the formula = [fasting insulin (μU/mL] × fasting glucose (mg/dL)/405]. HOMA-IR ≥ 2.5 was defined as having insulin resistance [34].

A part of the serum samples obtained from the collected blood samples was stored at −80 °C. Serum irisin, leptin, and adiponectin levels were measured at the Biochemistry Laboratories of the Faculty of Medicine at Marmara University with the ELISA method, using specific kits for each hormone. Irisin was measured using the Phoenix Pharmaceuticals Irisin (Human, Rat, Mouse) ELISA kit [35], leptin was measured using the Millipore Human Leptin ELISA kit [36], and adiponectin was measured using the Alpco Diagnostics Total Adiponectin ELISA and HMW & Total Adiponectin ELISA kits [37], based on the instructions provided by their manufacturers.

### 2.4. Statistical Analysis

The collected data were analyzed using the SPSS 20.0 (SPSS, Inc., Chicago, IL, USA) statistical package program. Mean, standard deviation, median, minimum, and maximum values of quantitative data, and frequency and percentage values of qualitative data, were calculated and tabulated. The Kolmogorov-Smirnov test was used for the obese and control groups separately, to test whether their data were normally distributed. Categorical data were analyzed using Pearson’s chi-squared tests, and normally distributed qualitative data were compared using Student’s *t*-test. The Mann-Whitney U and Kruskal-Wallis tests were used to compare non-normally distributed qualitative data. The Pearson correlation coefficient was used to investigate the relationships between variables. The threshold *p*-value for statistical significance was set at 0.05 [38].

## 3. Results

The ages of the children included in the study were in the range of 6–10 years, with a mean age of 8.4 ± 1.54. 42.5% of the children were male, while 57.5% were female. 

### 3.1. Anthropometric Parameters and Body Composition

The anthropometric parameters and body composition analysis results of the obese and control groups are given in Table 1. Values for weight, BMI, waist circumference, waist/height ratio, neck circumference, hip circumference, subscapular skinfold thickness, triceps skinfold thickness, muscle mass, body fat mass, and body fat ratio (%) were found to be significantly higher in the obese group (*p* < 0.05).

### 3.2. Metabolic Profile

While cholesterol, LDL cholesterol, 25 (OH) vit D3, calcium, phosphorus, ALP, and PTH values did not significantly differ between the two groups, fasting blood glucose, insulin, and HOMA-IR values were significantly higher in the obese group, whereas HDL and cholesterol values were significantly higher in the control group (*p* < 0.05) (Table 2).

According to their HOMA-IR values, 54.5% of the children in the obese group and 5.6% of those in the control group had insulin resistance. The difference between these rates was statistically significant (*p* = 0.000). Vitamin D was deficient in 13.6% of the children in the obese group, insufficient in 20.5%, and sufficient in 65.9%. Moreover, vitamin D was deficient in 5.6% of the children in the control group, insufficient in 30.5%, and sufficient in 63.9%. There was no significant difference between the groups in terms of their serum vitamin D levels (*p* = 0.348). 

### 3.3. Serum Leptin and Adiponectin Levels

The children in the obese group had significantly higher leptin levels and lower adiponectin levels than those in the control group (*p* < 0.05) (Table 1). Leptin and adiponectin levels did not differ based on sex or vitamin D classifications (*p* > 0.05). The mean leptin levels were 5.5 ± 3.28 ng/mL in the children without insulin resistance and 16.4 ± 8.25 ng/mL in those with insulin resistance (*p* = 0.000). The mean adiponectin levels were 8.0 ± 2.56 μg/mL in the children without insulin resistance and 6.2 ± 1.79 μg/mL in those with insulin resistance (*p* < 0.05).

### 3.4. Serum Irisin Serum Levels

**Figure 1:**
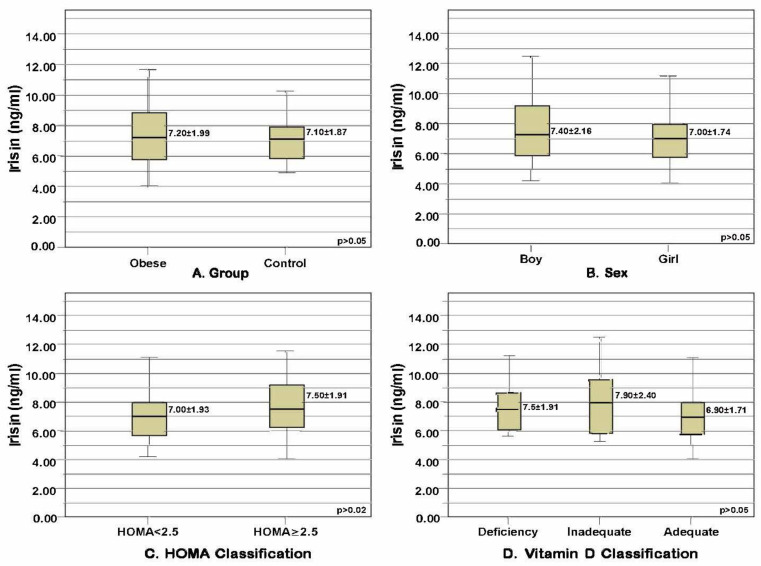
Mean and standard deviation values of irisin levels in the obese and control groups distributed based on certain parameters. (**A**) Serum irisin levels compared based on obesity status (*p* = 0.718, t = 0.363, *t*-test). (**B**) Serum irisin levels compared based on sex (*p* = 0.377, t = 0.888, *t*-test). (**C**) Serum irisin levels compared based on HOMA-IR classification (*p* = 0.277, t = −1.095, *t*-test). (**D**) Serum irisin levels compared based on vitamin D level classification (*p* = 0.311, χ^2^ = 2.336, Kruskal-Wallis test).

The mean serum irisin level of the children in the obese group (7.2 ± 1.99 ng/mL) and the mean serum irisin level of those in the control group (7.1 ± 1.87 ng/mL) were not significantly related to their sex, HOMA-IR, or vitamin D classification (*p* > 0.05) (Figure 1).

### 3.5. Correlations between Serum Irisin with Anthropometric Parameters, Body Composition, Metabolic Profile, Leptin, and Adiponectin

No statistically significant relationship was found between the serum irisin levels of the children and their anthropometric parameters, body composition analysis results, metabolic profile, leptin, or adiponectin values (*p* > 0.05) (Table 3). 

## 4. Discussion

The results of this case-control study demonstrated that serum irisin levels did not significantly differ between obese and normal-weight children. Irisin levels also did not have significant relationships with anthropometric parameters, body composition, metabolic profiles, leptin, or adiponectin.

In a meta-analysis investigating the relationship between irisin and obesity, overweight/obese individuals were generally determined to have higher irisin levels in circulation, although some of the examined studies provided different results. In subgroup analyses conducted based on ethnic origin, it was observed that irisin levels were higher in the obese group compared to the control group only in African participants, but there were no significant differences between the obese and control groups in European, Asian, or American samples [27]. It is suggested that elevated irisin levels in circulation in obesity are caused by the development of irisin resistance as an adaptive and compensatory response to metabolic dysfunction caused by obesity [39]. In this study, serum irisin levels did not significantly differ between the obese and control groups, similar to the results of other studies in the literature [18,19,20]. Moreover, in this study, there was no significant relationship between irisin and adiposity indicators (BMI, waist circumference, neck circumference, waist/height ratio, triceps skinfold thickness, subscapular skinfold thickness, fat mass (kg), fat ratio (%)) in the obese and control groups. The conflicting results of studies examining the relationship between irisin and obesity in children and adolescents may originate from differences in the ethnicities, ages, physiological parameters, and weight categories (normal weight or obese) of their participants.

Muscle mass is considered to be the main determinant of irisin levels in circulation under normal metabolic conditions, and skeletal muscle mass and irisin levels are expected to have a positive relationship [40]. Studies on the relationship between irisin and lean body mass revealed a positive relationship in Korean children [11] and a negative relationship in German children [41]. In this study, similar to the results of other studies [12,13,42,43], no significant relationship was found between irisin and muscle mass in obese and normal-weight children. The studies that found a relationship between irisin levels and muscle mass [11,41] were performed on adolescents, suggesting that puberty may be a potential determining factor in the relationship between irisin and muscle mass. The fact that the present study was conducted on prepubertal children, may explain the absence of a significant relationship between irisin and muscle mass parameters.

It was reported that irisin levels may differ between the sexes, as male and female children have different distributions and ratios of skeletal muscle, adipose tissue, and other body components [20]. Although Al-Daghri et al. [24] and Li Zhang et al. [15] determined higher levels of irisin in female children compared to male children, other studies have usually found no significant relationship between sex and irisin levels [11,12,16,18,44]. Likewise, in this study, irisin levels did not show a significant difference between male and female children. Additionally, like other results in the literature, there was no significant relationship found between age and irisin levels [17,18,21]. 

Irisin has potentially multiple favorable effects on glucose homeostasis and insulin resistance by increasing energy consumption, promoting glucose intake and glycogenolysis, and lowering gluconeogenesis [45]. In a meta-analysis of studies on the relationship between irisin and metabolic parameters in non-obese and non-diabetic adults, irisin levels were discovered to be positively and significantly associated with fasting blood glucose and HOMA-IR values [46]. On the other hand, in children, the relationship between irisin and parameters including insulin resistance and disrupted glucose metabolism has not been completely understood yet [10]. Al-Daghri et al. [24] showed a negative relationship between irisin levels and HOMA-IR values in female children. In contrast, a positive relationship between irisin levels, parameters of insulin resistance, and HOMA-IR has been identified [12,19,25]. In contrast to Different from these results, no significant relationship has been observed between irisin and glucose metabolism parameters in pediatric patients with type 2 diabetes and metabolic syndrome [47], both with or without insulin resistance [48], and severely obese children and adolescents [24]. In this study, although glucose parameter values were higher in the obese group compared to the control group, it was determined that irisin had no significant relationship to fasting blood glucose, insulin, or HOMA-IR in either group. Furthermore, irisin levels of the children who were included in this study did not differ depending on their status of insulin resistance. Despite the argument that irisin in circulation can modulate energy consumption in adults with altered/disrupted glucose metabolism, it was stated that this compensatory mechanism may be different, especially in preadolescent children who have a smaller muscle mass compared to adults [10].

It has been argued that irisin can influence lipid synthesis through steroidogenesis, and irisin secretion can be triggered in cases of hyperlipidemia to bring altered metabolism to its previous state [11]. On the other hand, in a meta-analysis of studies including non-obese and non-diabetic adults, irisin was not found to have any significant relationship to total cholesterol, HDL cholesterol, or LDL cholesterol [46]. Regarding children, the relationship between irisin levels in circulation and lipid profiles has been examined in various studies, and inconsistent findings have been obtained. Some studies on children revealed positive relationships between irisin and triglyceride, total cholesterol, and LDL cholesterol levels [11,12,19,44,49], whereas others showed negative relationships between irisin and triglyceride levels [17,21], and between irisin and HDL cholesterol levels [24]. However, Elizondo-Montemayor et al. [13] did not identify a relationship between irisin and lipid profiles. Similarly, in this study, no statistically significant relationship was found between the children’s lipid profiles and their irisin levels. Although the lipid profile parameter values of the obese children in this study were higher than those of the control group, the lack of a significant relationship between lipid profiles and irisin may be related to the lack of a significant relationship between irisin and obesity.

As both irisin and vitamin D are important modulators of the musculoskeletal system and energy homeostasis, in recent years, a relationship between vitamin D and irisin levels has been assumed [50]. The number of studies investigating this relationship in children is limited, and no significant relationship has been found between irisin and 25(OH) vitamin D levels in healthy children [51] or obese children [26]. Similarly, in this study, there was no significant relationship between the irisin levels of the children in both groups and their vitamin D levels. The lack of such a relationship may be associated with the fact that most of the children included in this study had sufficient vitamin D levels. Very little is known about the physiological relationship between irisin and vitamin D metabolism in children, and more clinical studies are needed to shed light on the underlying mechanisms.

There are few clinical studies on the relationship between irisin and adipokines in children, and their results are inconsistent [10]. Irisin levels in circulation and leptin levels were determined to have a positive relationship among Korean children [17], a negative relationship among Mexican children [21], and no significant relationship among Turkish children [25]. Irisin and adiponectin were found to show negative relationships in Italian [48] and Korean children [17], whereas no significant relationship was identified in Arab children [24]. In this study, irisin levels were also not significantly associated with leptin or adiponectin levels. Moreover, the obese children and those with insulin resistance in this study had higher leptin levels and lower adiponectin levels. These results were compatible with the relevant literature [52,53], suggesting that, compared to irisin, leptin and adiponectin may be more sensitive parameters in cases of obesity and insulin resistance.

This study had some limitations. First, our results came from a case-control design that did not allow causality statements. Second, the study had a relatively small sample size. Third, potential factors associated with irisin, such as exercise and diet, were not evaluated. Future studies with more potential factors and larger numbers of participants are needed to confirm our results. 

Despite some limitations, the strengths of this study include the fact that it examined irisin, anthropometric measurements, and body composition multidimensionally (circumference measurements, skinfold thickness, body composition analyses). Additionally, the relationships between irisin, leptin, adiponectin, and vitamin D, where there is limited information in children, were comparatively investigated. We believe that this study can add new evidence to the understanding of the relationships between irisin, adiponectin, leptin, and vitamin D, all of which are potential biomarkers that can be used in the diagnosis and treatment of childhood obesity.

## 5. Conclusions

In this study, it was determined that children in the obese group had high leptin levels and low adiponectin levels, and there was no significant difference between the irisin levels of the two groups. Moreover, irisin levels were not observed to have a significant relationship with anthropometric parameters, body composition analysis results, metabolic profiles, leptin, or adiponectin values in obese and normal-weight children. Further studies with larger samples examining potential factors associated with irisin levels are needed to determine the role of irisin in the pediatric population.

## Figures and Tables

**Table 1:** Anthropometric parameters and body compositions of the obese and control groups.

Parameters	Obese (*n* = 44)	Control (*n* = 36)	*t*	*p*
	$\bar{X}$ ± SD	$\bar{X}$ ± SD		
^a^ Age (years)	8.4 ± 1.48	8.3 ± 1.63	0.379	0.703
^a^ BMI (kg/m^2^)	23.6 ± 3.21	16.8 ± 2.22	11.244	0.000 *
^a^ Waist circumference (cm)	81.0 ± 8.82	62.2 ± 7.21	10.277	0.000 *
^b^ Neck circumference (cm)	31.2 ± 2.17	27.0 ± 1.59	−6.739	0.000 *
^a^ Hip circumference (cm)	86.7 ± 8.69	70.4 ± 7.31	8.959	0.000 *
^a^ Waist/height ratio	0.6 ± 0.05	0.5 ± 0.03	11.379	0.000 *
^a^ Triceps SFT (mm)	23.8 ± 3.43	13.2 ± 3.78	13.125	0.000 *
^a^ Subscapular SFT (mm)	18.1 ± 4.35	8.0 ± 2.80	12.074	0.000 *
^a^ Muscle mass (kg)	15.1 ± 3.39	12.1 ± 3.12	4.020	0.000 *
^a^ Fat mass (kg)	16.3 ± 5.35	5.4 ± 2.57	11.971	0.000 *
^a^ Fat ratio (%)	35.4 ± 7.52	18.0 ± 5.90	11.039	0.000 *

^a^ Student’s *t*-test; ^b^ Mann-Whitney U test; * *p* < 0.05, - *t*-Value is given for Student’s *t*-test, and Z-value is given for Mann-Whitney U test.

**Table 2:** Metabolic profiles of the obese and control groups.

Parameters	Obese (*n* = 44)	Control (*n* = 36)	*t*/Z	*p*
	$\bar{X}$ ± SD	$\bar{X}$ ± SD		
^b^ Fasting blood glucose (mg/dL)	92.2 ± 6.62	87.7 ± 7.10	2.931	0.004 *
^a^ Insulin (Iu/mL)	13.2 ± 9.12	6.2 ± 6.12	4.722	0.000 *
^a^ HOMA-IR	3.0 ± 2.12	1.4 ± 1.34	4.717	0.000 *
^a^ Triglyceride (mg/dL)	92.4 ± 46.02	60.1 ± 31.51	3.406	0.001 *
^b^ Cholesterol (mg/dL)	162.6 ± 23.80	166.9 ± 26.11	0.758	0.451
^b^ HDL cholesterol (mg/dL)	50.8 ± 10.85	64.3 ± 15.69	4.484	0.000 *
^a^ LDL cholesterol (mg/dL)	98.0 ± 21.97	94.4 ± 19.66	1.105	0.269
^a^ 25 OH vit D3 (ng/mL)	26.5 ± 16.54	23.5 ± 8.08	0.208	0.835
^b^ Ca (mg/dL)	9.7 ± 0.36	9.8 ± 0.29	0.845	0.401
^b^ P (mg/dL)	4.9 ± 0.50	4.9 ± 0.43	0.727	0.470
^a^ ALP (U/L)	227.0 ± 52.46	191.7 ± 59.07	1.058	0.290
^b^ Leptin (ng/mL)	14.6 ± 8.40	2.7 ± 2.38	4.424	0.000 *
^a^ Adiponectin (μg/mL)	6.3 ± 1.91	8.6 ± 2.50	−4.424	0.000 *

^a^ Student’s *t*-test; ^b^ Mann-Whitney U test; * *p* < 0.05; *t*-Value is given for Student’s *t*-test, and Z-value is given for Mann-Whitney U test.

**Table 3:** Correlations of the serum irisin levels of the obese and control groups with their anthropometric parameters, body composition, metabolic profile, leptin, and adiponectin values (r).

Parameters	Obese		Control	
	r	*p*	r	*p*
Age (years)	−0.212	0.166	−0.113	0.530
Body mass index (kg/m^2^)	−0.098	0.526	−0.012	0.947
Waist circumference (cm)	−0.197	0.200	−0.189	0.293
Neck circumference (cm)	−0.274	0.072	−0.037	0.839
Hip circumference (cm)	−0.138	0.372	−0.155	0.388
Waist/height ratio	−0.177	0.250	−0.074	0.682
Triceps SFT (mm)	−0.105	0.499	−0.282	0.111
Subscapular SFT (mm)	−0.212	0.168	−0.068	0.707
Muscle mass (kg)	−0.180	0.243	−0.017	0.926
Fat mass (kg)	−0.076	0.622	−0.263	0.139
Fat ratio (%)	−0.236	0.122	−0.279	0.116
Fasting blood glucose (mg/dL)	0.131	0.408	−0.058	0.746
Insulin (IU/mL)	0.114	0.478	−0.173	0.335
HOMA-IR	0.097	0.548	−0.173	0.336
Triglyceride (mg/dL)	0.131	0.410	−0.022	0.908
Cholesterol (mg/dL)	−0.126	0.428	0.106	0.559
HDL cholesterol (mg/dL)	−0.026	0.869	−0.039	0.828
LDL cholesterol (mg/dL)	−0.142	0.375	−0.044	0.811
Vitamin D (ng/dL)	0.160	0.298	−0.261	0.142
Calcium (Ca) (mg/dL)	0.233	0.142	−0.030	0.869
Phosphorus (P) (mg/dL)	−0.046	0.782	0.031	0.866
ALP (U/L)	0.019	0.933	−0.757	0.243
PTH (pg/mL)	0.065	0.732	−0.106	0.563
Leptin (ng/mL)	−0.006	0.969	−0.311	0.078
Adiponectin (μg/mL)	0.273	0.088	−0.035	0.847

## Data Availability

The data that support the findings of this study are available from the corresponding author, KE, upon reasonable request.
